# Conducting a Statewide Health Examination Survey: The Arkansas Cardiovascular Health Examination Survey (ARCHES)

**Published:** 2011-04-15

**Authors:** Namvar Zohoori, LeaVonne Pulley, Camille Jones, John Senner, Hylan Shoob, Robert K. Merritt

**Affiliations:** Chronic Disease Director and Associate Director of Science, Center for Health Advancement, Arkansas Department of Health; Fay W. Boozman College of Public Health, University of Arkansas for Medical Sciences, Little Rock, Arkansas; Arkansas Minority Health Commission, Little Rock, Arkansas; Arkansas Department of Health, Little Rock, Arkansas; Centers for Disease Control and Prevention, Atlanta, Georgia; Centers for Disease Control and Prevention, Atlanta, Georgia

## Abstract

**Introduction:**

The Arkansas Cardiovascular Health Examination Survey is a health and nutrition examination survey designed to serve as a demonstration project for collection of data on the prevalence of chronic diseases and their risk factors at the state level. The survey was conducted from mid-2006 through early 2008.

**Methods:**

We chose a cross-sectional representative sample of adult residents in Arkansas by using a 3-stage, cluster sample design. Trained interviewers conducted interviews and examinations in respondents' homes, collecting data on risk factors and diseases, blood pressure and anthropometric measurements, and blood and urine samples for analysis and storage. Food frequency questionnaires provided dietary and nutrient intake data. We accomplished the project using a collaborative model among several programs and partners within the state.

**Results:**

A total of 4,894 eligible households were contacted by telephone. Of these, refusals accounted for 2,748, and 2,146 gave initial consent to participate, for an initial response rate of 44%. The final number of completed household visits was 1,385, resulting in a final response rate of 28.3%.

**Conclusion:**

The Arkansas Cardiovascular Health Examination Survey is among the first state-level health and nutrition examination surveys to be conducted in the United States. By using a collaborative model and leveraging federal funds, we engaged several partners who provided additional resources to complete the project. The survey provides the state with valuable state-level data and information for program design and delivery.

## Introduction

States rely on the Behavioral Risk Factor Surveillance System (BRFSS) as the main source of state-level surveillance data. However, BRFSS is conducted via telephone interviews, collecting self-reported information. Self-reports do not present a complete picture of many chronic diseases, because self-report cannot provide information on undiagnosed disease or levels of control and respondent recall may be incomplete. Nationally, the National Health and Nutrition Examination Survey (NHANES) provides information on measured risk factors and diseases. However, these findings may not be applicable to individual states, and they do not influence local policy makers as much as do local data. There are no published reports of state-level health examination surveys, and to our knowledge the New York City Health and Nutrition Examination Survey is the only reported local example in the United States (1).

The Centers for Disease Control and Prevention (CDC) has provided funding to 4 states to conduct demonstration projects for the design, implementation, and completion of health examination surveys. Arkansas, Kansas, and Washington were funded in 2005, and Oklahoma was funded in 2007. To assess different approaches, CDC gave each state considerable freedom in methods by requiring only collection of data on blood pressure and cholesterol levels and adequate sampling of a designated priority population.

We report on the methods used in the Arkansas Cardiovascular Health Examination Survey (ARCHES) (Table 1). ARCHES collected data on a representative sample of noninstitutionalized adult Arkansans with oversampling of the black population, which was designated as our priority population because of its many health disparities in Arkansas. Interviews were conducted from mid-2006 through early 2008. ARCHES was a 1-time activity, with goals of 1) providing the Arkansas Department of Health (ADH) with data to implement population-based programs and policies for prevention and control of major chronic diseases and 2) serving as a demonstration project for a state-level health examination survey on the prevalence and risk factors for chronic diseases.

## Methods

### Funding, collaboration, and scope

**Figure 1:**
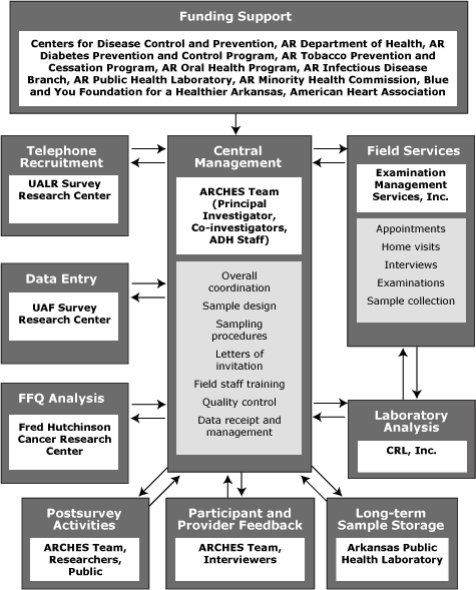
Arkansas Cardiovascular Health Examination Survey (ARCHES) collaboration model and flow of information. Abbreviations: AR, Arkansas; UALR, University of Arkansas at Little Rock; ADH, Arkansas Department of Health; UAF, University of Arkansas at Fayetteville; FFQ, food frequency questionnaire; CRL, Clinical Reference Laboratories.

The CDC Division for Heart Disease and Stroke Prevention provided initial funding for ARCHES ($760,000 over 2 years), through the ADH Heart Disease and Stroke Prevention program. To maximize the scope of ARCHES, we used this funding to encourage participation from several partners, which resulted in funding, donated materials, volunteer assistance, and collaboration from many programs within ADH and several external collaborators (Figure 1). Total cash available became $1.08 million. With this additional funding, we expanded ARCHES beyond its initial CDC mandate and included several questionnaire domains, covering many risk factors and health conditions, and the collection of examination data, including anthropometric measures and biological samples. The Science Review Council of the ADH and its institutional review board approved all protocols, instruments, procedures, consent forms, and other documents used for recruitment and data collection.

### Sample

ARCHES was a population-based, cross-sectional survey of noninstitutionalized adult (aged ≥18 y) residents in Arkansas, using a 3-stage clustered sample design (Table 1). In the first stage, we divided the 623 inhabited census tracts into 1) 188 tracts with a black population greater than 22.7% and 2) the remaining 435 tracts. We designated the first group, and the 5 largest tracts from the second group, as certainty clusters (ie, they were included in the sampling frame). We selected an additional 182 clusters from the remaining 430 tracts by using probability proportional to size, resulting in 375 sampled clusters. In the second stage, letters were sent to a random sample of households within the selected clusters, and then we randomly called households in each cluster until we had 4 households in that cluster that agreed to participate. In the third stage, in each selected household we determined the number of adults and sampled 1 person by using a computerized algorithm based on Kish sampling methods (2,3). If this person refused to participate, the next household on the list was called, until 4 respondents had agreed in each sampled cluster.

Our goal was to recruit approximately 1,500 participants, based on available funding and sample size calculations indicating that a sample of 1,344 would yield statistical power adequate to compare blacks and whites for several key cardiovascular variables. We verified the adequacy of the sample size by simulating results using BRFSS household data, Census 2000 racial data, and NHANES prevalence data.

We weighted the data in 2 steps to represent the Arkansas population. First, to account for the complex sampling plan, we computed a structural weight as the product of the inverse of the cluster sample probability (1 for certainty sample tracts, otherwise as computed by PROC SURVEYSELECT of SAS [SAS Institute, Inc, Cary, North Carolina]), households per sampled tract represented by each sample household, and adults per sampled household represented by each sample adult. Second, we accounted for nonresponse by using a postsampling weight that adjusted age, race, and sex categories to the 2007 Arkansas population estimates (4).

### Study population

Exclusion criteria were not speaking English and having psychiatric, cognitive, or developmental disorders, which were identified during the recruitment call. We excluded non-English speakers because, at the time of sampling, only 5% of the state's population were estimated to be Hispanic — not enough to form a significant part of the sample — and funding limitations did not allow oversampling of this subgroup.

We recruited participants through a letter followed by a telephone call. We first mailed letters and brochures (written at an average 7th-grade level) to selected households, explaining the survey and informing them that a telephone call would be made on behalf of ADH asking for participation of a person from the household. Within 2 weeks, we made calls to households to explain the survey, answer questions about it, and ask for enough household information to allow random selection of 1 person.

We briefly screened the selected person by telephone to ascertain eligibility, to answer the participant's questions about the survey, and to obtain initial verbal consent for participation. We then made an appointment with the selected person for an in-person interview and examination, at a location of the participant's choosing (in all cases, the home of the participant). We informed participants that blood and urine samples would be taken and asked them to fast overnight unless there were medical reasons not to. We also informed participants about benefits of participation, including the service that they would provide, provision of all laboratory test results (valued at approximately $260) to each participant, and gift cards of up to $50 ($40 for home visits and $10 for a returned Food Frequency Questionnaire [FFQ]).

### Data collection

To maximize participation and data completeness, we completed the interview, examination, and collection of blood and urine samples in 1 home visit. The only exception was the FFQ, which we left with the participant with instructions for completion and return to ADH, using self-addressed and stamped envelopes. Home visits ranged from 60 to 90 minutes, depending on skip patterns for questions and ease of anthropometric measurements and sample collection. Examination Management Services, Inc (EMSI) (Irving, Texas), a provider of specimen collection services for clinical trials and epidemiologic studies, collected all data.

Interviewers were nurses or other health professionals employed by EMSI and trained in phlebotomy and interviewing and examination techniques. In addition, we required that interviewers take training courses related to human subjects research and Health Insurance Portability and Accountability Act (HIPAA) privacy rules and participate in a 3-day training and certification session conducted by ARCHES investigators. Training included administering the questionnaire and standard protocols for drawing blood, measuring blood pressure, and performing anthropometric measurements. In accordance with Arkansas law, we also trained interviewers to follow required procedures if they observed child or adult abuse while in homes.

**Box. Major Content Areas of the Arkansas Cardiovascular Health Examination Survey Questionnaires and Physical Examination**

**Questionnaire Domains**

- General health and access to care
- Perceived stress
- Physical functioning (Activities of Daily Living and Instrumental Activities of Daily Living)
- Medical conditions and family medical history
- Diabetes
- Hypertension
- Knowledge of signs and symptoms of heart attack and stroke
- Cholesterol
- Aspirin use
- Oral health
- Physical activity
- Sleep disorders
- Fruit and vegetable consumption
- Self-reported weight and weight management
- Tobacco use and exposure
- Alcohol consumption
- Occupation
- Social support and depression
- Demographic information and housing
- Health insurance
- Food security
- Complementary and alternative medicine use
- Reactions to race
- Hepatitis C risk factors
- List of all medications and supplements
- Food Frequency Questionnaire

**Physical Examination**

- Weight
- Height
- Abdominal circumference
- Arm circumference
- Pulse (3 readings throughout interview)
- Blood pressure (3 readings throughout interview)

**Blood and Urine Tests**

- Blood chemistry panel (alanine, albumin, alkaline phosphatase, aminotransferase, aspartate aminotransferase, bicarbonate, direct/indirect bilirubin, calcium, chloride, cholesterol, creatinine phosphokinase, creatinine, gamma-glutamyl transpeptidase, iron, phosphate, lactate dehydrogenase, lipase, magnesium, potassium, sodium, total bilirubin, total protein, and triglycerides)
- Complete blood count (hematocrit, hemoglobin, mean corpuscular hemoglobin, mean corpuscular hemoglobin concentration, mean corpuscular volume, red blood cell count, white blood cell count, and differential counts)
- Calcium
- Cystatin C
- Fasting glucose
- Fasting serum insulin
- Hemoglobin A1c (for known diabetics only)
- High sensitivity C-reactive protein
- Homocysteine
- Parathyroid hormone
- Serum cotinine
- Urinary albumin-to-creatinine ratio

The ARCHES questionnaire consisted of up to 285 questions (depending on skip patterns) covering behavioral, psychosocial, socioeconomic, and demographic variables, personal and family medical history, cardiovascular and other chronic disease risk factors, health care access, and other subjects (Box). Questions were mainly from BRFSS (5) and NHANES (6). The complete questionnaire was pretested through cognitive interviews with a convenience sample of low-income community volunteers, and necessary changes were made. Nutrient intake data were collected by using the FFQ developed by the Nutrition Assessment Shared Resource (7) of the Fred Hutchinson Cancer Research Center (FHCRC). Questionnaires are available on the ARCHES page of the ADH website (http://www.healthy.arkansas.gov/programsServices/chronicDisease/Initiatives/ Pages/Arches.aspx).

After the participant provided written informed consent, interviewers administered the questionnaire and recorded responses on paper forms. Interviewers also examined medicine bottles for all medications (prescribed and over-the-counter, including dietary supplements), and recorded medication names. We measured participants' height, weight, and abdominal circumference by using standard NHANES protocols, while they wore light clothing and no shoes. We used a Tanita digital, self-calibrating scale (model HD-351) (Tanita Corporation of America, Inc, Arlington Heights, Illinois) to measure weight. After measurement of arm circumference and use of an appropriate-sized cuff, interviewers recorded blood pressure 3 times at intervals during the interview process by using standard protocols (6), with an Omron HEM-907XL monitor (Omron Healthcare, Inc, Bannockburn, Illinois). For each recording, the monitor recorded the average of 3 separate readings, for up to 9 readings and 3 recordings.

After administering the questionnaire and taking anthropometric measurements, interviewers collected blood and urine samples. Interviewers placed the samples in containers with frozen-gel bags, processed them in the field according to protocols provided by Clinical Reference Laboratories, Inc (CRL) (Lenexa, Kansas), and shipped them to CRL, a laboratory certified according to the CDC Lipid Standardization Program (http://www.cdc.gov/labstandards/lsp.html), for analysis and reporting. The laboratory also froze aliquots of blood and urine and shipped them to ADH for storage and future analyses.

We managed all activities, including tracking of interviews, movement of forms and biological samples, data entry, and quality control, centrally at ADH, through contracts with several external entities (Figure 1). After completing the interview, EMSI personnel shipped questionnaires, forms, and biological samples to CRL, where blood and urine samples were further processed and analyzed. CRL sent the results of analyses, along with forms and questionnaires, to ADH, where they were logged and entered in the main database. The University of Arkansas at Fayetteville Survey Research Center scanned the main questionnaires, digitized the data, and transmitted them electronically to ADH. ADH personnel logged and shipped the FFQs that had been mailed back by participants to FHCRC for analysis. FFQ results were electronically transmitted back to ADH. After all data were received and entered into the main database, we created an analysis dataset, stripped of personal identifiers, for use in further analyses.

We took several steps to ensure data integrity and quality. One of the senior investigators telephoned a 5% random subsample of respondents within a few days of the interviews to ascertain interviewer accuracy and find out about participants' experiences with the interviewers. We reported inaccuracies or problems to EMSI for corrective action. Before scanning and data entry, we hand-checked all questionnaires for readability, correct skip patterns, and missing data, and made corrections to the extent possible (calling respondents when needed). We pulled a 5% random subsample of paper copies of the main questionnaires and checked them against the electronic data to ensure accuracy in digitization. We hand-checked all FFQs before scanning and analysis. Laboratory analyses were subject to internal laboratory standards and checks by CRL and to range and consistency checks of all data by ARCHES staff.

Interviewers informed respondents with high blood pressure of their measurements and advised them to seek appropriate medical care. We monitored the results of blood and urine tests as they were received by ARCHES; respondents with results above predetermined critical values were contacted immediately and advised to seek medical attention, with an offer to fax results directly to their health care provider. We sent all blood and urine results, within 2 weeks of receipt, to each respondent along with a cover letter, with abnormal values flagged for attention and discussion with their health care provider.

## Results

**Figure 2:**
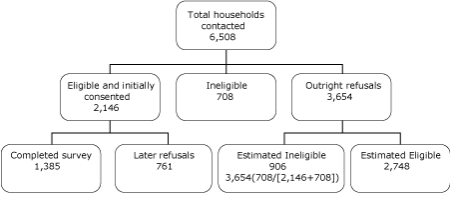
Response rate calculation (using Council of American Survey Research Organizations formula) for Arkansas Cardiovascular Health Examination Survey sample. The response rate was the number who completed surveys (1,385) divided by the sum of the number who were eligible and initially consented (2,146) plus the estimated number who were eligible among those who refused (2,748): 1,385/(2,146 + 2,748) = 28.3%.

Of the 6,508 households contacted by telephone, 4,894 were eligible. Of these, refusals accounted for 2,748, and 2,146 gave initial consent to participate, for an initial response rate of 44%. The final number of completed household visits was 1,385, resulting in a CASRO (Council of American Survey Research Organizations) response rate of 28.3% (Figure 2).

Among completed visits, 1,265 (91.3%) participants also mailed in their FFQs, and 1,202 (86.8%) gave consent to freezing biological samples for future analyses. Also, 1,115 (80.5%) consented to future contact for follow-up surveys.

Compared with the state population, the ARCHES sample had a higher proportion of women (66.9% vs 51.7%), a higher proportion of blacks (23.8% vs 14.9%), and an older age distribution (Table 2). Median annual household income for the sample, about $35,000, was just slightly lower than the state median income of about $36,600 in 2007 (8), the time when the sample was developed.

## Discussion

In organizing ARCHES, we had 3 goals: 1) to foster collaboration among programs, 2) to leverage CDC funds, and 3) to minimize the burden on resources of the health department. We achieved the first 2 goals by approaching a number of internal programs and external partners and encouraging their participation. This resulted in additional cash funding from 8 entities (Figure 1), with a 1.42:1 ratio of total cash available to that provided by CDC. We achieved the third goal by contracting with different entities to carry out much of the work, such as initial recruitment, setting appointments, conducting interviews and examinations, data entry, and laboratory analyses (Figure 1). Using this approach, each completed survey cost about $780. However, as a potential future ongoing surveillance effort, efficiency can be improved by using a different approach, focusing on higher priority data collection, and eliminating sample storage.

The tasks of overall coordination, data management, and quality control were maintained by the staff at ADH, directed by the principal investigator and the co-investigators — a collaboration between ADH, the Fay W. Boozman College of Public Health at the University of Arkansas for Medical Sciences, and the Arkansas Minority Health Commission. This aspect of ARCHES, the ability to partner with many entities, was a major accomplishment of the project and a factor in its successful completion.

We completed interviews and examinations on 1,385 people, exceeding the goal of 1,344. Our sample was older and had a higher proportion of women than the Arkansas adult population (9). This is not surprising for a household survey (10) and was probably further influenced by our use of landline telephones only. At the time of sampling, cellular telephone numbers linked to addresses at census tract level (our primary sampling unit) were not publicly available; therefore, we were compelled to use landlines. Also, our BRFSS survey in 2007 was landline-based, facilitating eventual comparison of ARCHES results with BRFSS. The higher proportion of blacks in ARCHES was by design, fulfilling the CDC requirement of oversampling 1 priority population. All 3 of these differences were taken into account in the individual survey sampling weights, and the use of analytic software that allows accommodation for these sampling factors. Approximately one-third of those giving initial consent were either unable to be scheduled or declined to be scheduled for the in-home visit. This rate is close to that of the national REGARDS study, which used similar methods (11). Although the 28% CASRO response rate may be of some concern, there are no similar published state-level studies for comparison. However, the acceptable range of individual sampling weights (0.23-3.08) and the similar household incomes of participants and nonparticipants increase our confidence in the representativeness of the weighted sample.

ARCHES has provided ADH with a large amount of data that are being used to generate much-needed health information. Some of this information, such as levels of undiagnosed or uncontrolled disease (eg, hypertension, diabetes) and risk factors (such as overweight and obesity), is available for the first time at the state level. The data differ greatly from those obtained from self-reported BRFSS surveys. For example, obesity, hypertension, and diabetes are 50% to 55% more prevalent than indicated by BRFSS data (unpublished data). The data provide the ADH programs, researchers, and clinicians with information necessary to address the state's worsening public health problems. Already the data have been used to inform policy makers and legislators about the more prevalent chronic diseases in the state and have resulted in a legislative request for an interim study of hypertension in Arkansas. The data are also being used by ADH, public health researchers, and students to analyze specific patterns of consumption, risk factors, and diseases in the state, leading to program development and improvement. In addition, the bank of frozen biological specimens will be used for future studies of risk factors and diseases in the state.

Conducting a state-level health examination survey was challenging. Arkansas's model shows that by working with internal and external partners, and by contracting out major survey tasks, it is possible to conduct such a health examination survey without putting undue burden on the human and financial resources of the health department. The local information provided can be of benefit in addressing chronic diseases at the state level. This benefit, however, can be fully realized only if such surveys are repeated regularly, allowing states to track changes and effects of policies and programs. On the basis of our experience with ARCHES, we believe that repeating such surveys approximately every 5 years is feasible and has the potential to provide timely information for monitoring progress toward intermediate and long-term goals related to outcomes.

## Figures and Tables

**Table 1:** Timeline of Contact With Participants and Main Activities of the Arkansas Cardiovascular Health Examination Survey (ARCHES)

**Stage of Survey**	Mode of Contact With Participant	Activities Conducted (Dates Accomplished)	Organization and Personnel Involved
Sampling stage 1	NA	Selected 375 of 623 census tracts and oversampled tracts with highest proportions of blacks. (May 2006)	ADH, ARCHES staff, and statistician
Sampling stage 2	Letters to random sample of households within selected clusters	Informed about the survey, included informational brochure, and told to expect telephone call. (June 2006-October 2007)	ADH, ARCHES staff
Sampling stage 3	Initial telephone calls to households in random order	Provided additional information and answered questions, obtained initial household consent, selected participating adult, obtained participant's initial verbal consent to participate; maximum 4 per cluster. (June 2006-February 2008)	University of Arkansas at Little Rock, Survey Research Center
Field work	Calls to participants to make appointment for interview	Made appointment for home visit, provided additional information about the interview and exam, and gave instructions about fasting for blood draw. (July 2006-March 2008)	Examination Management Services, Inc, call center
	Home visits	Obtained written informed consent, completed questionnaire, performed anthropometric and blood pressure measurements, and collected blood and urine samples. (July 2006-March 2008)	Examination Management Services, Inc, interviewers
Laboratory analysis and data entry	NA	Analyzed blood and urine samples and transmitted electronically to ADH. (July 2006-April 2008)	Clinical Reference Laboratories, Inc
		Scanned and digitized main questionnaire data and transmitted electronically to ADH. (July 2006-July 2008)	University of Arkansas at Fayetteville, Survey Research Center
		Scanned and analyzed food frequency questionnaire data and transmitted electronically to ADH. (July 2006-July 2008)	Fred Hutchinson Cancer Research Center
	Telephone calls to some participants	Verified data for quality control purposes and to complete missing or discrepant data. (July 2006-March 2008)	ADH, ARCHES staff
Reporting to participants	Telephone calls to some participants	Called participants with critical values within 24 hours of receipt of results. (July 2006-March 2008)	ADH, principal investigator
	Letters to all participants	Included copy of all blood and urine results, thank-you letter and general explanation of results, with instructions for further follow-up with primary care provider. Also included gift cards. (July 2006-May 2008)	ADH, ARCHES staff

Abbreviation: NA, not applicable; ADH, Arkansas Department of Health.

**Table 2:** Demographic Characteristics of the Arkansas Cardiovascular Health Examination Survey (ARCHES) Sample and of the Arkansas Adult Population, 2007

Characteristic	No. of ARCHES Respondents (%)	2007 Arkansas Adult Population, %^a^
**Sex**		
Men	459 (33.1)	48.3
Women	926 (66.9)	51.7
**Race**		
White	1,056 (76.2)	85.1
Black	329 (23.8)	14.9
**Age, y**		
18-49	430 (31.0)	57.0
50-64	540 (39.0)	24.2
≥65	415 (30.0)	18.8

a Source: National Center for Health Statistics (4).
